# Repurposing lipid-lowering drugs on asthma and lung function: evidence from a genetic association analysis

**DOI:** 10.1186/s12967-024-05359-5

**Published:** 2024-07-03

**Authors:** Yue Zhang, Zichao Jiang, Lingli Chen, Ting Lei, Xiangrong Zheng

**Affiliations:** 1 Department of Pediatrics, Xiangya Hospital, Central South University, Hunan, 410008 China; 2 Department of Orthopaedics, Xiangya Hospital, Central South University, Hunan, 410008 China

**Keywords:** Lipids, Lowering-lipid therapy, Asthma, Mendelian randomization, Casual association

## Abstract

**Objective:**

To explore the correlation between asthma risk and genetic variants affecting the expression or function of lipid-lowering drug targets.

**Methods:**

We conducted Mendelian randomization (MR) analyses using variants in several genes associated with lipid-lowering medication targets: HMGCR (statin target), PCSK9 (alirocumab target), NPC1L1 (ezetimibe target), APOB (mipomersen target), ANGPTL3 (evinacumab target), PPARA (fenofibrate target), and APOC3 (volanesorsen target), as well as LDLR and LPL. Our objective was to investigate the relationship between lipid-lowering drugs and asthma through MR. Finally, we assessed the efficacy and stability of the MR analysis using the MR Egger and inverse variance weighted (IVW) methods.

**Results:**

The elevated triglyceride (TG) levels associated with the APOC3, and LPL targets were found to increase asthma risk. Conversely, higher LDL-C levels driven by LDLR were found to decrease asthma risk. Additionally, LDL-C levels (driven by APOB, NPC1L1 and HMGCR targets) and TG levels (driven by the LPL target) were associated with improved lung function (FEV1/FVC). LDL-C levels driven by PCSK9 were associated with decreased lung function (FEV1/FVC).

**Conclusion:**

In conclusion, our findings suggest a likely causal relationship between asthma and lipid-lowering drugs. Moreover, there is compelling evidence indicating that lipid-lowering therapies could play a crucial role in the future management of asthma.

**Supplementary Information:**

The online version contains supplementary material available at 10.1186/s12967-024-05359-5.

## Introduction

Asthma is a chronic lung disease characterized by airway inflammation and constriction during attacks. Its typical symptoms include wheezing, chest tightness, and coughing [1]. So far, the incidence of asthma has been attributed to a combination of environmental and genetic factors. However, the specific mechanism of asthma attacks has not yet been fully clarified [2]. Approximately 3 billion people worldwide suffer from asthma, and this number is projected to increase to 10 billion by 2025 [3]. The symptoms of asthma can vary in severity from mild to severe, and some individuals may experience life-threatening symptoms [4]. A significant body of research has demonstrated that persistent wheezing is associated with slower lung function improvement during adolescence [5]. Moreover, individuals with persistent asthma are more likely to experience an increased incidence of other respiratory diseases, such as bacterial pneumonia, which can be induced by an increased nasopharyngeal carriage of Streptococcus pneumoniae [6]. Asthma and its related complications have posed a significant public health challenge, leading to high morbidity and, in severe cases, a high fatality rate [7]. Unfortunately, asthma cannot currently be cured, but it can be effectively managed with current medical care [8]. While asthma cannot be cured, it can be effectively managed with appropriate therapy, allowing individuals to maintain a healthy condition [9]. The traditional treatment for controlling acute asthma attacks involves the use of inhaled glucocorticoids and β-agonists [10]. Some asthma patients benefit from the combination of inhaled glucocorticoids (GC) with inhaled β-agonists. However, several patients are unable to control their condition despite receiving large doses of inhaled GC or even oral GC, a condition known as GC-resistant asthma [11]. Therefore, it is important to explore new drugs for the treatment of asthma.

Although there is increasing technological advancement in drug research, drug repurposing has garnered more attention [12, 13]. Drug repurposing, also known as drug repositioning, involves using existing drugs for new therapeutic purposes [14, 15]. Compared to developing new drugs, drug repurposing offers reduced development time, higher approval rates, and existing safety data [16]. This approach has been employed in many diseases, such as psoriasis [17], COVID-19 [18, 19], HPV-associated cervical cancer [20], endometrial cancer [21], and tubulointerstitial fibrosis [22], among others.

Lipid-lowering drugs comprise a range of medications used to reduce blood cholesterol levels, thereby lowering the risk of heart disease and stroke [23]. The primary types of lipid-lowering drugs include statins, alirocumab, ezetimibe, mipomersen, evinacumab, fenofibrate, acipimox, and volanesorsen [24]. Previous studies have suggested that statins may have therapeutic effects on diseases beyond their lipid-lowering function, such as Alzheimer’s disease [25]. In humans, statins have been shown to reduce the risk of lung cancer [26]. Interestingly, statins and fibrates have been found to reduce host inflammation [27].Moreover, statins have also shown potential in the treatment of asthma [28]. An observational study discovered an association between statin use and fewer hospitalizations for asthma attacks in asthma patients [29]. A double-blind study found that statins improve the anti-inflammatory efficacy of inhaled corticosteroids in asthma patients [30]. Another study found that among patients with severe asthma, those who took statins achieved better asthma control [31]. Although the benefits of statins for asthma have been widely reported, less evidence has been shown regarding the role of other lipid-lowering drugs in asthma. Hence, we aimed to explore the association between different lipid-lowering drugs and asthma.

Mendelian Randomization (MR) is a technique for investigating the causal influence of a modifiable exposure on disease using genetic variation in genes [32]. In MR research, genetic variants serve as instrumental variables for the exposure of interest, under the assumption that they are randomly assigned at conception and unaffected by confounding variables [33]. This approach enables researchers to address some of the limitations of conventional observational studies, such as confounding and reverse causality [34]. Therefore, we conducted a two-sample MR analysis in this study to explore the potential causal effect of lipid-lowering drugs on asthma.

## Methods

### Study design and source of GWAS Summary dataset

**Fig. 1:**
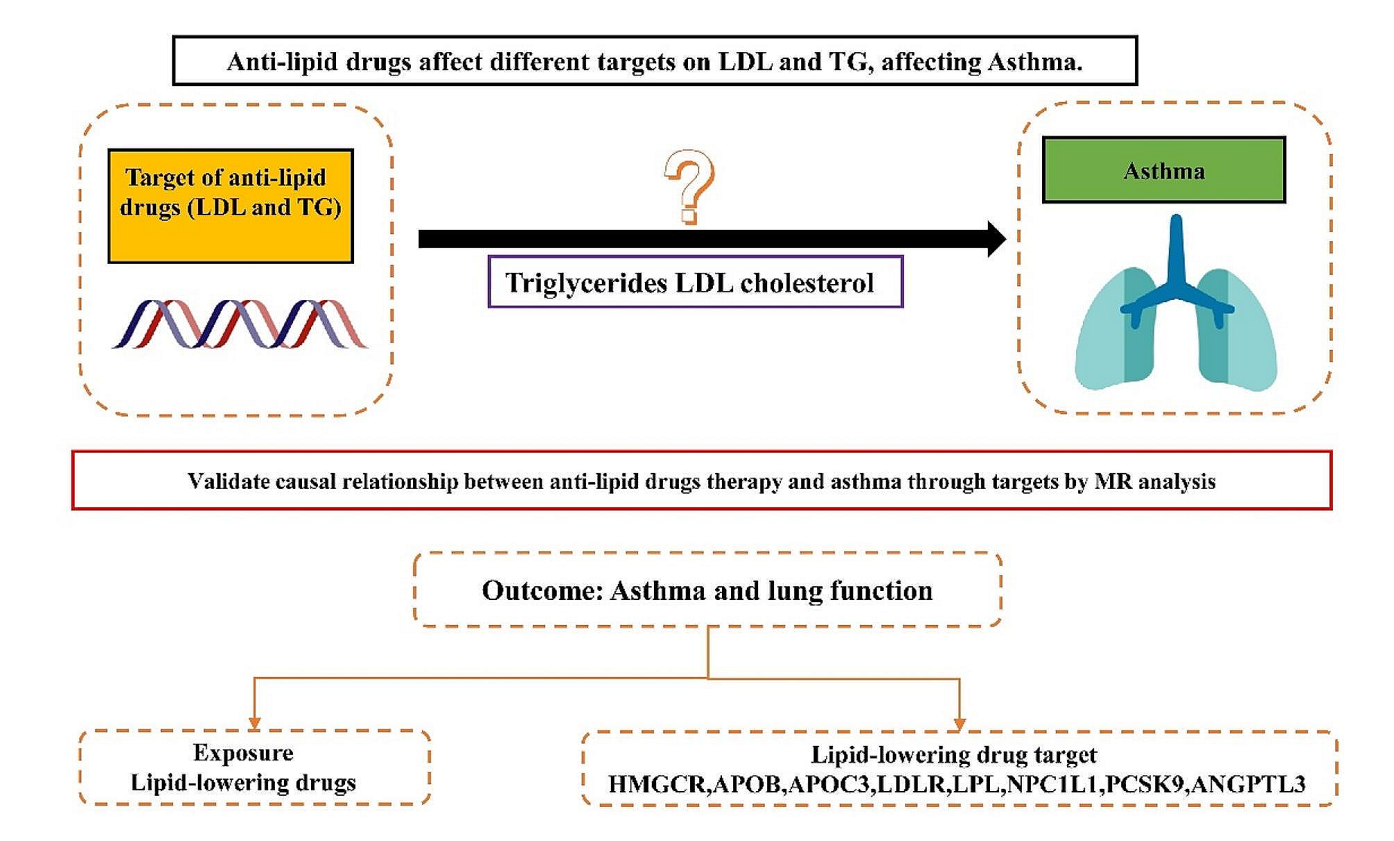
Schematic and flowchart progress of lipid-lowering drugs and asthma

In our study, we conducted a two-sample MR analysis focusing on genetic variations located in or near genes encoding the relevant medication targets, as illustrated in Fig. 1 [35]. The lipid traits were extracted from the Global Lipids Genetics Consortium (GLGC, http://lipidgenetics.org/), which included Low-Density Lipoprotein Cholesterol (LDL-C), triglycerides (TG), and total cholesterol (TC). Additionally, we selected four asthma datasets with the largest sample sizes and three lung function datasets from the GWAS summary database, primarily comprising European populations. The Data Ark provides access to GWAS summary statistics in the GWAS-VCF format, facilitating the efficient storage of genetic variants, annotations, and metadata. The platform includes GWAS datasets from various consortia, such as those imported from the EBI database and those developed for MR-Base. Researchers can access scripts for working with GWAS summary statistics uploaded by Shea Andrews. According to the note in the GWAS database, lung function was defined as the “FEV1/FVC” ratio. This ratio is calculated using spirometry, a technique that analyzes how much air you can forcibly exhale from your lungs. FEV1 (forced expiratory volume in one second) measures the volume of air expelled in a single second, while FVC (forced vital capacity) measures the total amount of air forcibly exhaled in one breath. These datasets are derived from Genome-Wide Association Studies (GWAS), which are observational studies of a genome-wide set of genetic variants in different individuals to determine if any variant is associated with a trait. Moreover, we collected a dataset for coronary heart disease (PMID:26,343,387, Population: mixed, cases: 60,801, controls: 123,504) from the IEU website (https://gwas.mrcieu.ac.uk/) as a positive control outcome. The detailed information is presented in Table 1.

**Table 1:** The baseline information of GWAS summary data related to LDL-C, triglyceride, coronary heart disease, asthma and lung function

Datasets	Source or IEU ID	PMID	Population	Case (*n*)	Control (*n*)
**LDL-cholesterol**	GLGC	24,097,068	Mixed	188,577	
**Triglycerides**	GLGC	24,097,068	Mixed	188,577	
**Coronary heart disease**	CARDIoGRAMplusC4D	26,343,387	Mixed	60,801	123,504
**Asthma**	ukb-a-66	NA	European	39,049	298,110
	ebi-a-GCST90014325	34,103,634	European	56,167	352,255
	ebi-a-GCST90038616	33,959,723	European	56,087	428,511
	ebi-a-GCST90018795	34,594,039	European	38,369	411,131
**Lung function**	ebi-a-GCST007431	30,804,560	European	321,047	
	ebi-a-GCST90025978	34,226,706	European	371,898	
	ebi-a-GCST90029026	29,892,013	European	446,811	

GLGC: Global Lipids Genetics Consortium (http://lipidgenetics.org/); CARDIoGRAMplusC4D: Coronary ARtery DIsease Genome Wide Replication and Meta-analysis (CARDIoGRAM) plus The Coronary Artery Disease (C4D) Genetics (http://www.cardiogramplusc4d.org/)

### Target identification of lipid-lowering drug and MR analysis

Firstly, we selected several commonly used lipid-lowering drugs for investigation, including statins, alirocumab, ezetimibe, mipomersen, evinacumab, and volanesorsen. Next, we searched the DrugBank (https://go.drugbank.com/) and ChEMBL (https://www.ebi.ac.uk/chembl/) websites to identify the targets of these drugs. Following the intersection of these target results, we selected six targets, including HMG-CoA reductase (HMGCR, Statins), Subtilisin/kexin type 9 (PCSK9, Alirocumab), Niemann-Pick C1-like protein 1 (NPC1L1, Ezetimibe), Apo-B 100 mRNA (APOB, Mipomersen), Angiopoietin-related protein 3 (ANGPTL3, Evinacumab), and Apolipoprotein C-III mRNA 3’UTR (APOC3, Volanesorsen).The low-density lipoprotein receptor (LDLR) is a key protein that regulates cholesterol levels in the body [36]. Lipoprotein lipase (LPL) is a crucial enzyme in lipid metabolism and transport [37]. Therefore, two essential targets, LDLR and LPL, were included in our study. Further details are shown in Table 2.

Lipid-lowering drugs play a crucial role in managing cholesterol levels and reducing the risk of coronary heart disease (CHD) [38]. Therefore, to enhance the credibility of the targets, we considered CHD as a positive control to validate the targets. If the targets were validated, they could undergo further analysis. To determine if lipid-lowering drugs had a causal impact on asthma, we employed several methods, including the inverse variance weighted (IVW) technique, the MR Egger method, the weighted median method, and the weighted mode method. Single-nucleotide polymorphisms (SNPs) were selected as instrumental variations (IV) from genome-wide association study (GWAS) summary data to be used as a genetic tool in MR research. Additionally, the IVs had to meet the following criteria: (1) they were highly correlated with the exposure, (2) they were not associated with confounders, and (3) they affected outcomes only through the exposure. SNPs were selected based on the conditions of *P* < 5e-8, r2 < 0.001, and kb = 100.

**Table 2:** Target gene information of lowering-lipid drugs derived from different drug-gene interaction databases

Therapies	Drug class	Target protein (Encoding gene)	Databases	Selected
**Lowering LDL**				
	**Statins**	3-hydroxy-3-methylglutaryl-coenzyme A reductase (HMGCR)	DrugBank	**HMGCR**
		Integrin alpha L(ITGAL)	DrugBank	
		Histone deacetylase 2(HDAC2)	DrugBank	
		HMG-CoA reductase (HMGCR)	ChEMBL	
	**Alirocumab**	Proprotein convertase subtilisin/kexin type 9(PSCK9)	DrugBank	**PSCK9**
		Subtilisin/kexin type 9(PCSK9)	ChEMBL	
	**Ezetimibe**	Niemann-Pick C1-like protein 1(NPC1L1)	DrugBank	**NPC1L1**
		Sterol O-acyltransferase 1(SOAT1)	DrugBank	
		Aminopeptidase N(ANPEP)	DrugBank	
		Niemann-Pick C1-like protein 1(NPC1L1)	ChEMBL	
	**Mipomersen**	mRNA of ApoB-100(APOB)	DrugBank	**APOB**
		Apo-B 100 mRNA (APOB)	ChEMBL	
	**Evinacumab**	Angiopoietin-related protein 3(ANGPTL3)	DrugBank	**ANGPTL3**
		Angiopoietin-related protein 3(ANGPTL3)	ChEMBL	
	**Fenofibrate**	Peroxisome proliferator-activated receptor alpha (PPARA)	DrugBank	**PPARA**
		Peroxisome proliferator-activated receptor alpha (PPARA)	ChEMBL	
	**Acipimox**	Non		
		Non		
		LDL Receptor (LDLR)		**LDLR**
**Lowering TG**	**Evinacumab**	Angiopoietin-related protein 3(ANGPTL3)	DrugBank	**ANGPTL3**
		Angiopoietin-related protein 3(ANGPTL3)	ChEMBL	
	**Fenofibrate**	Peroxisome proliferator-activated receptor alpha (PPARA)	DrugBank	**PPARA**
		Peroxisome proliferator-activated receptor alpha (PPARA)	ChEMBL	
	**Volanesorsen**	Apolipoprotein C-III(APOC3)	DrugBank	**APOC3**
		Apolipoprotein C-III mRNA 3’UTR(APOC3)	ChEMBL	
		Lipoprotein Lipase (LPL)		**LPL**

DrugBank: DrugBank online (https://go.drugbank.com/drugs); ChEMBL: ChEMBL Database (https://www.ebi.ac.uk/chembl/)

### Sensitivity analysis

We utilized the MR Egger and inverse variance weighted (IVW) methods to assess the effectiveness and stability of the MR analysis between exposures and outcomes. A P-value of 0.05 indicates strong heterogeneity in results from different populations, which can be demonstrated by the heterogeneity I2 using a percentage description. Subsequently, the MR analysis was rerun after removing outlier SNPs. Finally, to illustrate the trend and stability of the data, four plots were created: a scatter plot, a forest plot, the leave-one-out test, and a funnel plot. A scatter plot illustrates how single nucleotide polymorphisms (SNPs) influence exposure and outcome variables, helping visualize the relationship between genetic variations and exposure and outcomes of interest. It shows how changes in the exposure variable correspond to changes in the outcome variable based on genetic data. A forest plot is used to display the results of MR analysis, especially when examining the effects of multiple exposures on a single outcome, or vice versa. It allows for a visual comparison of effect sizes and confidence intervals among different exposures or outcomes, aiding in the identification of significant associations and providing a clear summary of the MR results. The leave-one-out plot is used for sensitivity analysis, systematically removing one SNP at a time from the MR analysis to assess the robustness of the results. By observing how each SNP influences the overall causal estimate, researchers can evaluate the impact of specific genetic variants on the MR analysis. A funnel plot is employed to assess asymmetry in MR analysis, which can indicate potential biases or reliability issues. It helps identify publication bias, small-study effects, and other sources of bias in MR studies. Funnel plots can suggest an overrepresentation of small studies with significant results, highlighting potential weaknesses in the MR analysis. R software and associated R packages were used for all MR analyses. All the MR analyses were performed using the Two Sample MR (version 0.5.10), Mendelian Randomization (version 0.8.0), and MRPRESSO package (1.0) in R Software 4.3.2 (https://www.R-project.org). The meta-analysis was performed using the meta package. All the used R package could be found on the website (cran.r-project.org/web/), and also, these package, GWAS summary data, along with the code could be acquired from authors with request.

## Results

### Positive control MR analysis

**Fig. 2:**
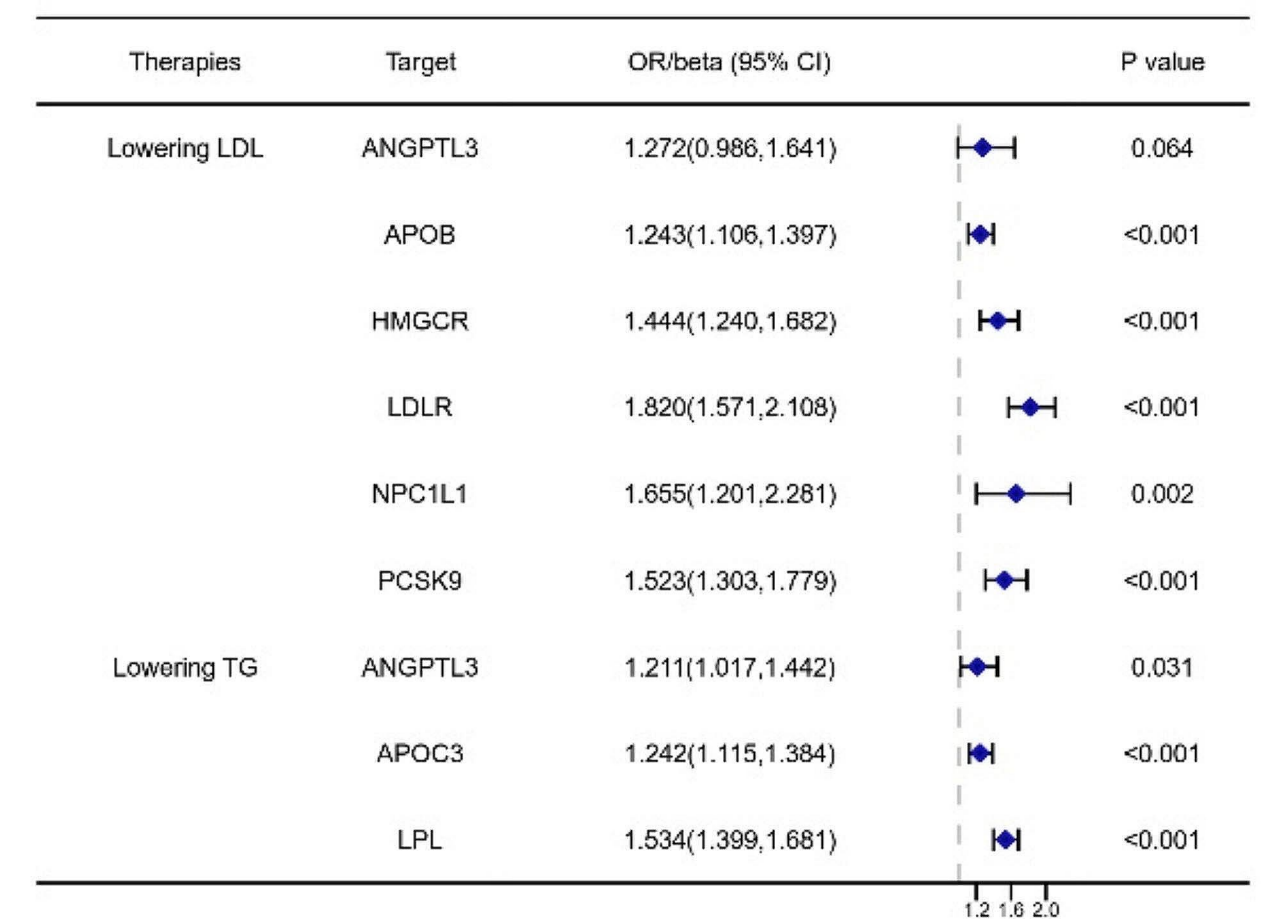
The forest plot for the lipid-lowering target with coronary heart disease (CHD). (CI: confidence interval; OR: odds ratio.)

Before conducting the MR analysis between lipid-lowering therapy and asthma, a positive control MR analysis was performed to assess the effectiveness of lipid-lowering drugs on coronary heart disease (CHD), a well-established fact. This analysis aimed to verify whether lipid-lowering drugs were indeed effective in treating CHD. The results of the MR analysis on different lipid-lowering drugs and their impact on CHD are summarized in Table 3; Fig. 2. The analysis showed that genetic variants associated with increased LDL levels, driven by the APOB, HMGCR, NPC1L1, and PCSK9 genes, were associated with an increased risk of CHD (APOB: OR = 1.243, 95%CI: 1.106, 1.397; HMGCR: OR = 1.444, 95%CI: 1.240, 1.682; NPC1L1: OR = 1.655, 95%CI: 1.201, 2.281; PCSK9: OR = 1.523, 95%CI: 1.303, 1.779). Similarly, an increased risk of CHD was observed with exposure to drugs targeting APOC3 (OR = 1.242, 95%CI: 1.115, 1.384). Furthermore, genetic variants associated with LDLR (OR = 1.820, 95%CI: 1.571, 2.108) and LPL (OR = 1.534, 95%CI: 1.399, 1.681) were also associated with an increased risk of CHD. The sensitivity analysis of the MR analysis between lowering-LDL and lowering-TG drugs with CHD showed no significant heterogeneity for the HMGCR, PCSK9, NPC1L1, ANGPTL3, and LDLR targets (Table S1).

**Table 3:** The MR analysis of the effect of different lowering LDL-cholesterol and lowering triglyceride on coronary heart disease

Therapies	Targets	F	SNP(*n*)	Beta (95%CI)	Se	OR (95%CI)	*p*
**Lowering LDL-C**							
	ANGPTL3	133.2343	3	0.240(-0.014,0.495)	0.130	1.272(0.986,1.641)	0.064
	APOB	220.2499	19	0.217(0.101,0.334)	0.060	1.243(1.106,1.397)	< 0.001
	HMGCR	151.0792	7	0.368(0.215,0.520)	0.078	1.444(1.240,1.682)	2.18E-06
	LDLR	117.1812	10	0.599(0.452,0.746)	0.075	1.820(1.571,2.108)	1.38E-15
	NPC1L1	94.5279	3	0.504(0.183,0.825)	0.164	1.655(1.201,2.281)	0.002
	PCSK9	130.3063	10	0.421(0.265,0.576)	0.079	1.523(1.303,1.779)	1.18E-07
	PPARA		0				
	LPL		0				
**Lowering TG**							
	ANGPTL3	209.4853	3	0.240(-0.014,0.495)	0.130	1.272(0.986,1.641)	0.064
	APOC3	298.7056	10	0.217(0.108,0.325)	0.055	1.242(1.115,1.384)	8.74E-05
	LPL	200.0503	22	0.428(0.336,0.519)	0.047	1.534(1.399,1.681)	6.56E-20
	LDLR		0				
	PPARA		0				

F: F statistics; SNP: single nucleotide polymorphism; Beta: beta coefficient; CI: confidence interval; OR: odds ratio

### The effect of different lipid-lowering drugs on asthma and lung function (FEV1/FVC)

**Fig. 3:**
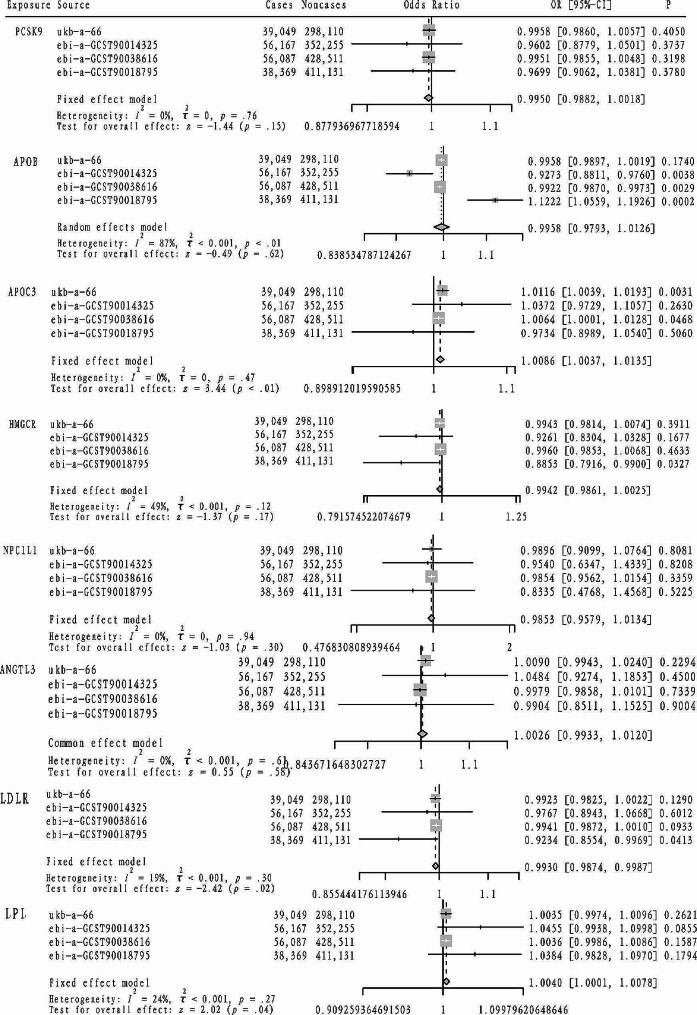
The casual effect of different lipid-lowering therapies on asthma risk was detected using the mendelian randomization method and the meta-analysis method was used to pool the MR analysis results of each lowering therapy. (Square: each OR value; Rhomboid: the pooled OR value of fixed effect model; CI: confidence interval; OR: odds ratio)

**Fig. 4:**
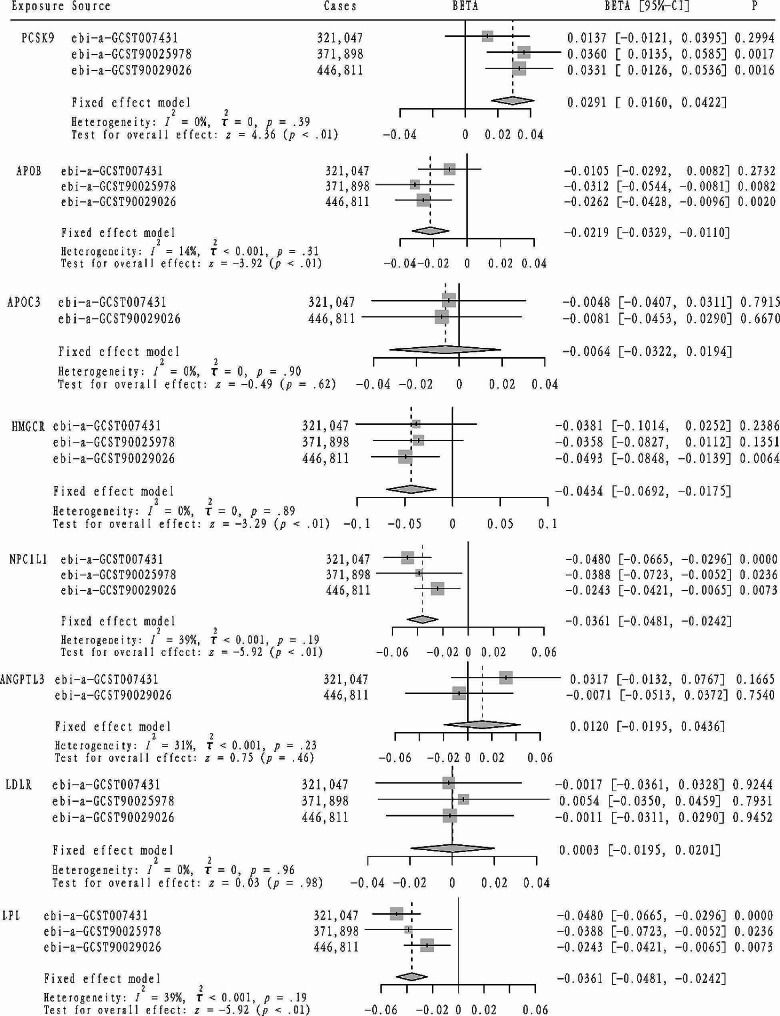
The causal effect of different lipid-lowering therapies on FEV1/FVC risk was detected using the mendelian randomization method, and the meta-analysis method was used to pool the mendelian randomization analysis results of each lowering therapy. (Square symbols: each beta value, Rhomboid symbols: the pooled beta value of the fixed-effect model. CI: confidence interval.)

As indicated in Table 2, seven targets were initially considered for analysis: HMGCR, PCSK9, NPC1L1, APOB, ANGPTL3, PPARA, and APOC3. Additionally, due to their significance in lipid metabolism control, LDLR and LPL targets were also examined. Initially, coronary heart disease was used as a positive control outcome to assess the effectiveness of this approach, with the analysis results depicted in Fig. 1. Based on these validated targets, further analysis was conducted. Subsequently, as shown in Fig. 3, an elevated TG level driven by the APOC3 target was found to increase the risk of asthma (OR = 1.0086, 95%CI: 1.0037–1.0135). Similarly, an increased TG level driven by LPL was associated with an increased risk of asthma (LPL: OR = 1.0040, 95%CI: 1.0001–1.0078). However, an elevated LDL-C level driven by LDLR was found to decrease the risk of asthma (LDLR: OR = 0.9930, 95%CI: 0.9874–0.9987). Furthermore, lung function(FEV1/FVC), as depicted in Fig. 4, showed that an increased LDL-C level driven by the APOB, HMGCR, and NPC1L1 targets led to improve lung function (FEV1/FVC) (APOB: beta=-0.0219, 95%CI: -0.0329, -0.0110; HMGCR: beta=-0.0434, 95%CI: -0.0692, -0.0175; NPC1L1: beta=-0.0361, 95%CI: -0.0481, -0.0242). Similarly, an increased TG level driven by the LPL target was associated with a decrease in lung function (FEV1/FVC) (beta=-0.0361, 95%CI: -0.0481, -0.0242).

### Sensitivity analyses

The sensitivity analysis for the MR analysis was conducted using the MR Egger and inverse variance weighted (IVW) methods, demonstrating the robustness and low heterogeneity of the MR analysis results (Supplementary Tables 15 and Supplementary Table 16). Additionally, the scatter plot, forest plot, leave-one-out plot, and funnel plot illustrated the stability of the MR analysis results (Supplementary Figs. 1–8). The scatter plot illustrates the relationship between the exposure variable (lipid-lowering medicines) and the outcome variable (asthma or FEV1/FVC) using instrumental variables (Supplementary Fig. 1.5). The forest plot graphically depicts the effect estimates and confidence intervals for each SNP (Supplementary Figs. 2 and 6). Furthermore, the leave-one-out analysis revealed that no single nucleotide polymorphism (SNP) had a substantial effect on the causal inference (Supplementary Figs. 3 and 7). This suggests that the overall causal link identified between lipid-lowering medicines and a lower risk of asthma or FEV1/FVC was not influenced by any single SNP, underscoring the robustness of the findings. Additionally, the funnel plot and the MR Egger regression test showed no indication of asymmetry, indicating the absence of directional horizontal pleiotropy (Supplementary Figs. 4 and 8).

## Discussion

Our study is the first to investigate the causal association between asthma and lipid-lowering drugs using MR. The MR analysis provided compelling evidence for a positive association of APOC3 (OR = 1.0086, 95% CI = 1.0037–1.0135) and LPL (OR = 1.0040, 95% CI = 1.0001–1.0078) with asthma risk, and a negative association of LDLR (OR = 0.9930, 95% CI = 0.9874–0.9987) with asthma risk. Additionally, in terms of lung function, an increased LDL-C level driven by the APOB, HMGCR, and NPC1L1 targets resulted in a reduction in lung function (FEV1/FVC) (APOB: beta = -0.0219, 95% CI: -0.0329, -0.0110; HMGCR: beta = -0.0434, 95% CI: -0.0692, -0.0175; NPC1L1: beta = -0.0361, 95% CI: -0.0481, -0.0242), while an increased TG level driven by the LPL target also led to a reduction in lung function (FEV1/FVC) (beta = -0.0361, 95% CI: -0.0481, -0.0242). These findings suggest that lipid-lowering drugs may have a significant role in the future treatment of asthma.

Asthma is one of the most common chronic diseases affecting both children and adults, characterized by complex gene-environment interactions [39]. It is characterized by recurrent airway obstruction and bronchial hyperresponsiveness, with symptoms such as wheezing, coughing, chest tightness, and shortness of breath [2].Almost 200,000 Americans and over 30 billion people globally suffer with asthma [40]. Asthma affects nearly 200,000 Americans and over 30 billion people globally [40], with an estimated 2.5 million deaths attributed to asthma each year [41]. Patients with severe asthma, in particular, face greater challenges as traditional drugs may not be effective [42]. Therefore, the development of new asthma treatments is crucial. However, traditional drug discovery methods typically take 12–17 years and cost $2–3 billion to bring a new drug to market [43]. These methods also have a high risk of failure due to the complexity and uncertainty of developing new compounds [44]. Consequently, there is growing interest in drug repurposing, which explores the potential of existing drugs for treating new diseases. Drug repurposing offers advantages such as shorter development timelines, higher approval rates, and lower overall development costs compared to traditional drug discovery methods [45].

The relationship between asthma and lipid metabolism has been extensively researched. Asthmatic patients often exhibit significantly higher levels of sputum LTC4, LTD4, and LTE4 than healthy individuals [46]. A meta-analysis has shown that patients with asthma tend to have higher LDL and total serum cholesterol levels compared to non-asthmatic individuals [47]. Similarly, higher TG/high-density lipoprotein cholesterol (HDL-C) ratios are associated with a higher prevalence of asthma [48]. Research suggests that low blood high-density lipoprotein cholesterol levels in children are linked to an increased risk of asthma in adolescence [49]. Moreover, high triglyceride levels have been linked to elevated levels of exhaled nitric oxide (FeNO) and aeroallergen sensitivity in 7-year-old children [50]. Given the close association between lipid metabolism and asthma, various drugs targeting the lipid pathway have been developed, which have shown potential in relieving asthma symptoms [51]. However, these new drugs are either expensive or have not yet been proven safe. Lipid-lowering drugs are medications designed to decrease blood cholesterol levels [38]. Statins, Alirocumab, Ezetimibe, Mipomersen, Evinacumab, Fenofibrate, Acipimox, and Volanesorsen are among the most common lipid-lowering drugs. Therefore, we aim to investigate the relationship between these lipid-lowering drugs and asthma using MR analysis.

Consistent with previous studies, we found that an increased TG level driven by the APOC3(OR = 1.0086, 95%CI: 1.0037–1.0135) and LPL (OR = 1.0040, 95%CI: 1.0001–1.0078) target were associated with an increased risk of asthma. Several studies have found an association between elevated serum triglyceride levels and the presence of asthma, particularly in patients with obesity [52, 53]. Furthermore, a study reported that serum TGs were significantly higher in patients with asthma, even after adjusting for factors such as BMI, blood eosinophils, and statin use [52].Volanesorsen is a novel antisense oligonucleotide inhibitor of APOC3 mRNA developed for the treatment of familial chylomicronemia syndrome [54, 55]. However, although the MR analysis indicated that the Volanesorsen showed statistically significant effect in asthma risk, the effective OR of this drug was 1.0026, which is so small that it may not manifest significant effect in clinical application. This finding may indicate a potential clinical advice that Volanesorsen may be a preferential choice for lowering-lipids treatment for patients with asthma risk or history of asthma. In addition, the real effect of this target in the asthma risk need further experiment demonstration, and our finding could be only identified as a pioneer study.

However, contrary to previous studies, we also observed that an increased LDL-C level driven by LDLR led to a decreased risk of asthma (LDLR: OR = 0.9930, 95%CI: 0.9874–0.9987). Some studies found that asthma was more prevalent in the high-risk groups for elevated LDL cholesterol, suggesting an association between higher LDL levels and increased asthma risk [48].However, LDL-C is not a homogeneous particle, which consists of several distinct subclasses that vary in size, density, and atherogenic potential. Nicola et al. reported that LDL-1 levels were comparable between the asthma group and the healthy subjects’ group (56 ± 16% vs. 53 ± 11, p = NS), whereas LDL-2 was significantly lower in asthmatics compared to controls (35 ± 8% vs. 43 ± 10%, *p* = 0.0074) [56].Research suggests that LDLR plays a role in asthma by negatively regulating airway hyperreactivity and goblet cell hyperplasia through an apo E-LDLR pathway, which acts as a natural negative regulator of these asthma-related processes [57]. Activation of this pathway, potentially through apo E mimetic peptides, could offer a novel treatment approach for asthma patients. Moreover, LDLR has been associated with suppressing IgE production and the expression of Th2 and Th17 cytokines in asthma models [58]. These findings underscore the potential therapeutic implications of targeting the apo E-LDLR pathway in managing asthma symptoms and inflammation.

Furthermore, we found that an increased LDL-C level driven by the APOB, HMGCR, NPC1L1 target resulted in a reduction in lung function (FEV1/FVC) (APOB: beta=-0.0219, 95%CI: -0.0329, -0.0110; HMGCR: beta=-0.0434, 95%CI: -0.0692, -0.0175; NPC1L1: beta=-0.0361, 95%CI: -0.0481, -0.0242). Statins, which are considered the first-line treatment for high cholesterol, work by blocking the HMG CoA reductase enzyme [59]. Due to their various impacts on the inflammatory process, statins may have potential therapeutic benefits for asthma treatment [60]. According to Amir et al., statins have demonstrated anti-inflammatory, anti-remodeling, and immunomodulatory properties that may benefit asthma patients by enhancing lung function and reducing airway hyper-reactivity [61]. Rosuvastatin treatment has been shown to improve lung pathology by suppressing cytokine production mediated by Th2 and Th17 cells [62].Furthermore, rosuvastatin also affects airway hyperresponsiveness, lung inflammation, and oxidative stress [63]. Research has shown that rosuvastatin can alleviate airway inflammation and oxidation by affecting NOS and reducing pro-inflammatory cytokines and inflammatory cells [64]. Studies have shown that simvastatin can decrease the levels of IL-4 and IL-5 in bronchoalveolar lavage fluid [65].Simvastatin prevented airway remodeling in asthma at an early stage [66]. Moreover, there is scant direct data from earlier studies regarding the connection between ezetimibe and mipomersen with asthma. According to our findings, APOB mipomersen, statins, and ezetimibe show great potential for improving lung function. Additionally, Haldar et al. describe a novel subset of patients with “obesity asthma.” These patients exhibit distinct clinical characteristics, such as late-onset asthma, severe symptoms, and poor response to inhaled corticosteroids, among others [67]. Similarly, the study discovered that obese asthmatics had a higher risk of hospitalization than lean asthmatics [68]. Hence, we believe that these lipid-lowering drugs may benefit obese asthmatic patients. Lipid-lowering drugs show great potential in treating obese asthmatic patients. However, further experimental investigation will be necessary to assess their specific efficacy in the future.

Our study has several strengths. Firstly, it is the first MR study to investigate the relationship between lipid-lowering medications and asthma. Secondly, MR can help mitigate limitations inherent in traditional observational studies, such as reverse causality and confounding, thus enhancing the robustness of our findings. Additionally, we used four of the largest asthma databases for our meta-analysis, which increased the reliability of our conclusions due to the large sample size. Furthermore, we employed appropriate effect models based on heterogeneity, using the “random effects” model for strong heterogeneity and the “fixed effects” model otherwise. We also conducted a sensitivity analysis to ensure the validity of our results. However, there are several limitations to our study. Firstly, our study was limited to a European population due to data restrictions in GWAS, which limits the generalizability of our findings to other populations. Secondly, due to the application limitation of MR analysis, the MR analysis cannot assess the effects of long-term exposure to lipid-lowering drugs or the effects of each subtype of drug. Thirdly, we were unable to explore dose-response relationships between these drugs and asthma. Cell and animal experiments, as well as prospective clinical studies, are necessary to investigate these aspects further in future. Furthermore, our study only looked at how individual drugs affect asthma, and the effects of combining different lipid-lowering drugs need to be investigated. Finally, aside from MR, multi-omic-based and network-based approaches are common methods used to explore new indications for existing drugs in an orderly manner [69]. Hence, we hope to employ multi-omic-based and network-based approaches in the future to investigate the relationship between lipid-lowering drugs and asthma, aiming to enhance the reliability and robustness of our results. In conclusion, our study provides evidence supporting a potential causal relationship between asthma and lipid-lowering drugs. The findings suggest that lipid-lowering drugs may have a significant impact on reducing asthma symptoms, indicating that lipid-lowering therapies could be crucial in the future management of asthma.

## Conclusion

In this study, we found that TG levels driven by the APOC3, and LPL targets were associated with an increased risk of asthma. However, we also found that LDL-C levels driven by the LDLR target were associated with a decreased risk of asthma. Additionally, LDL-C levels (driven by APOB, NPC1L1 and HMGCR targets) and TG levels (driven by the LPL target) were associated with improved lung function (FEV1/FVC). LDL-C levels driven by PCSK9 were associated with decreased lung function (FEV1/FVC). These findings underscore the potential importance of lipid-lowering therapies in the future management of asthma. However, further clinical trials are needed to confirm the effects of lipid-lowering medications on asthma. Additionally, further experimental research is required to elucidate the underlying mechanisms.

### Electronic supplementary material

Below is the link to the electronic supplementary material.

Supplementary Material 1

Supplementary Material 2

Supplementary Material 3

Supplementary Material 4

Supplementary Material 5

Supplementary Material 6

Supplementary Material 7

Supplementary Material 8

Supplementary Material 9

Supplementary Material 10

## Data Availability

The lipid traits were extracted from the Global Lipids Genetics Consortium (GLGC, http://lipidgenetics.org/), including LDL-C, TG and TC. Furthermore, we chose four asthma datasets with the biggest sample sizes and three lung function datasets, with most the dataset’s population being Europeans. Moreover, we collected a coronary heart disease dataset (PMID:26343387, Population: mixed, case:60,801, control:123,504) from the IEU website (https://gwas.mrcieu.ac.uk/) as a positive control outcome.

## Abbreviations

HMGCR: HMG-CoA reductase
PCSK9: Proprotein Convertase Subtilisin/Kexin Type 9
NPC1L1: NPC1 Like Intracellular Cholesterol Transporter 1
APOB: Apolipoprotein B
ANGPTL3: Angiopoietin Like 3
PPARA: Peroxisome Proliferator Activated Receptor Alpha
APOC3: Apolipoprotein C3
LDLR: Low Density Lipoprotein Receptor
LPL: Lipoprotein Lipase
MR: Mendelian randomization
IVW: Inverse variance weighted
TG: Triglyceride
GLGC: The Global Lipids Genetics Consortium
SNPs: Single-nucleotide polymorphisms
IV: Instrumental variations
CHD: Coronary heart disease
TyG: Triglyceride-glucose index
HDL-C: High-density lipoprotein cholesterol
