# Supplementary figures and images for: Repurposing lipid-lowering drugs on asthma and lung function: evidence from a genetic association analysis
Source: J Transl Med. 2024 Jul 3;22:615. doi: 10.1186/s12967-024-05359-5 (PMC11223406; doi:10.1186/s12967-024-05359-5)

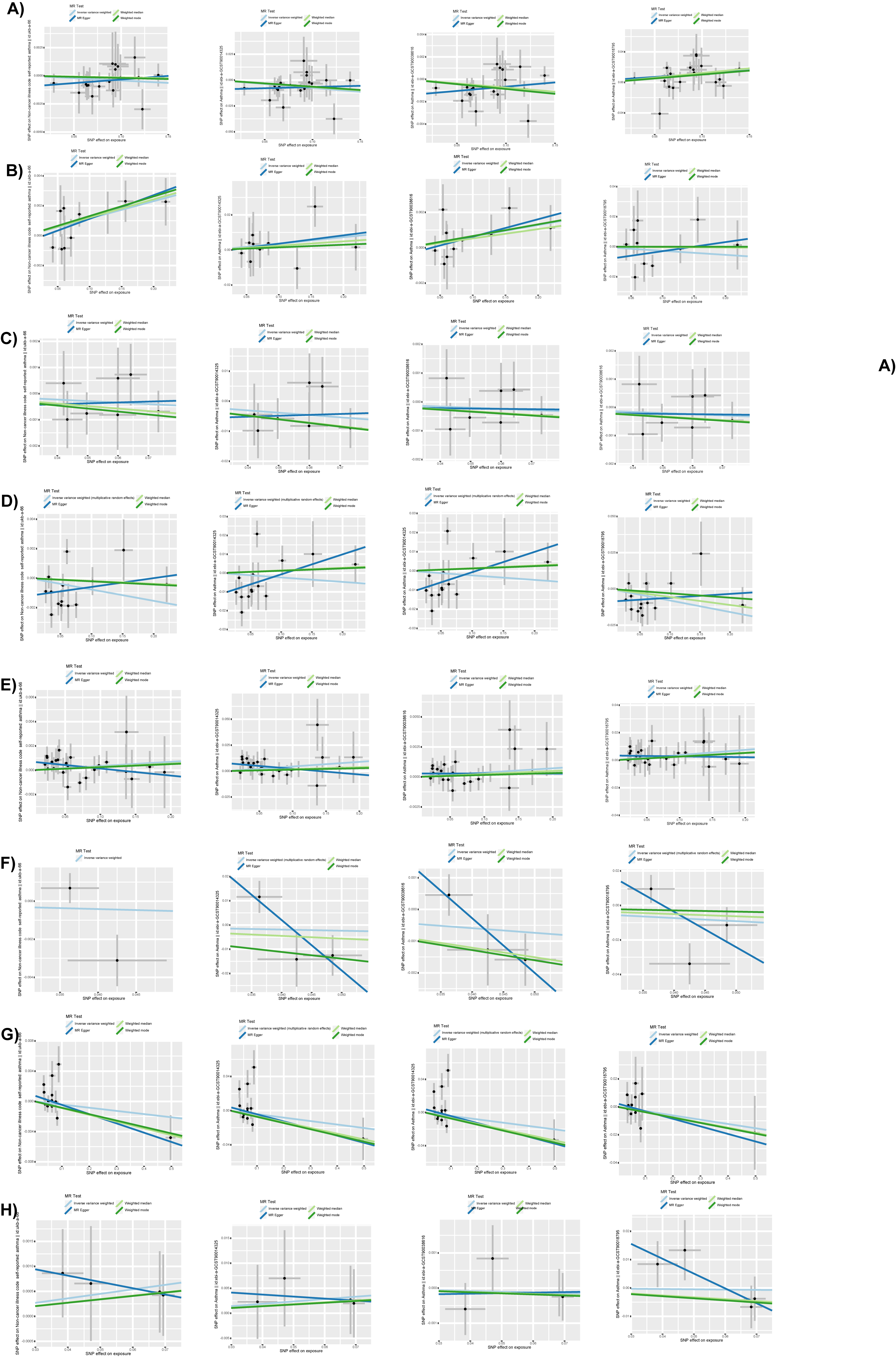

Supplement: Supplementary file 1 — Supplementary Material 1 [file 12967_2024_5359_MOESM1_ESM.png]

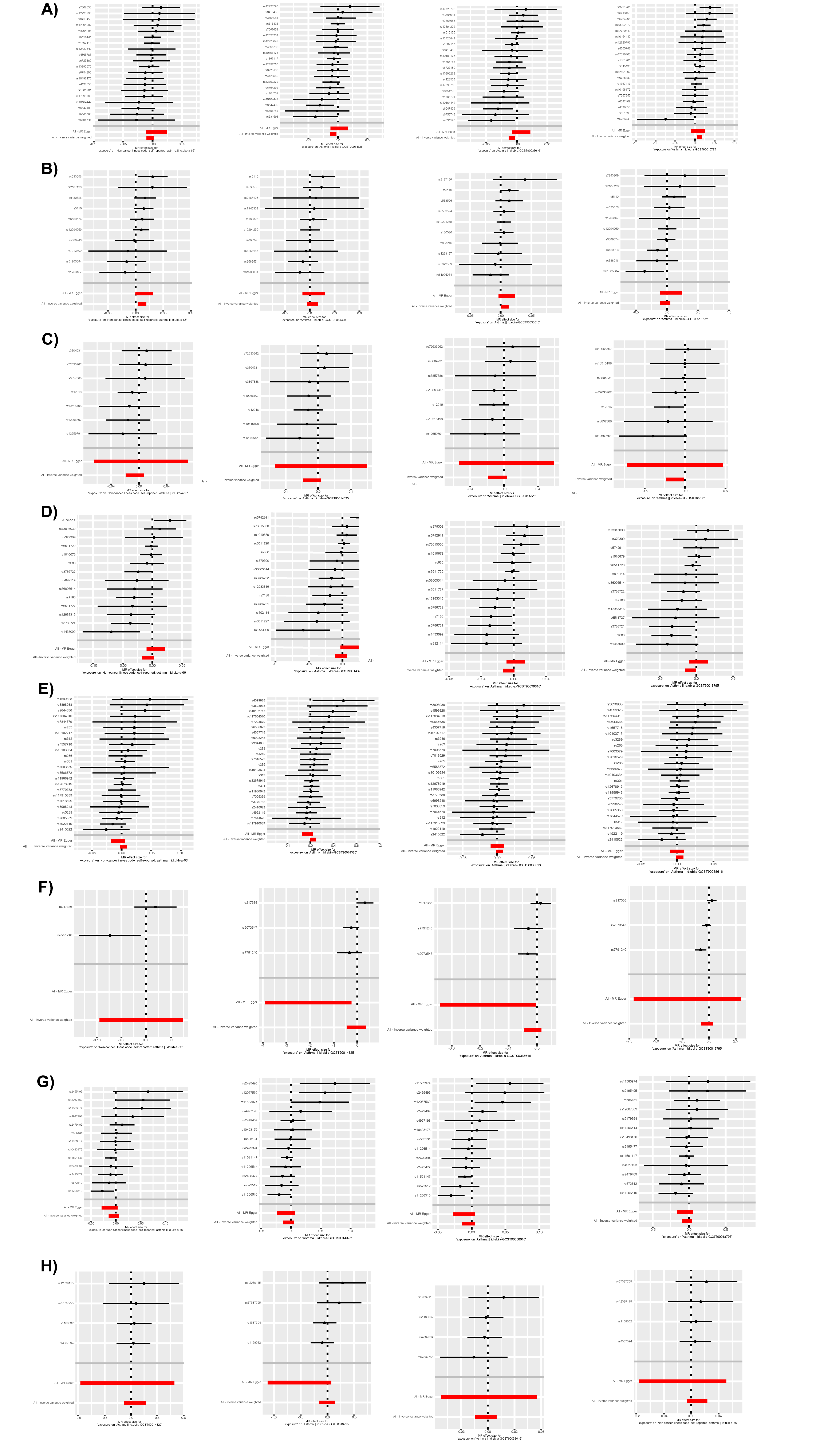

Supplement: Supplementary file 2 — Supplementary Material 2 [file 12967_2024_5359_MOESM2_ESM.png]

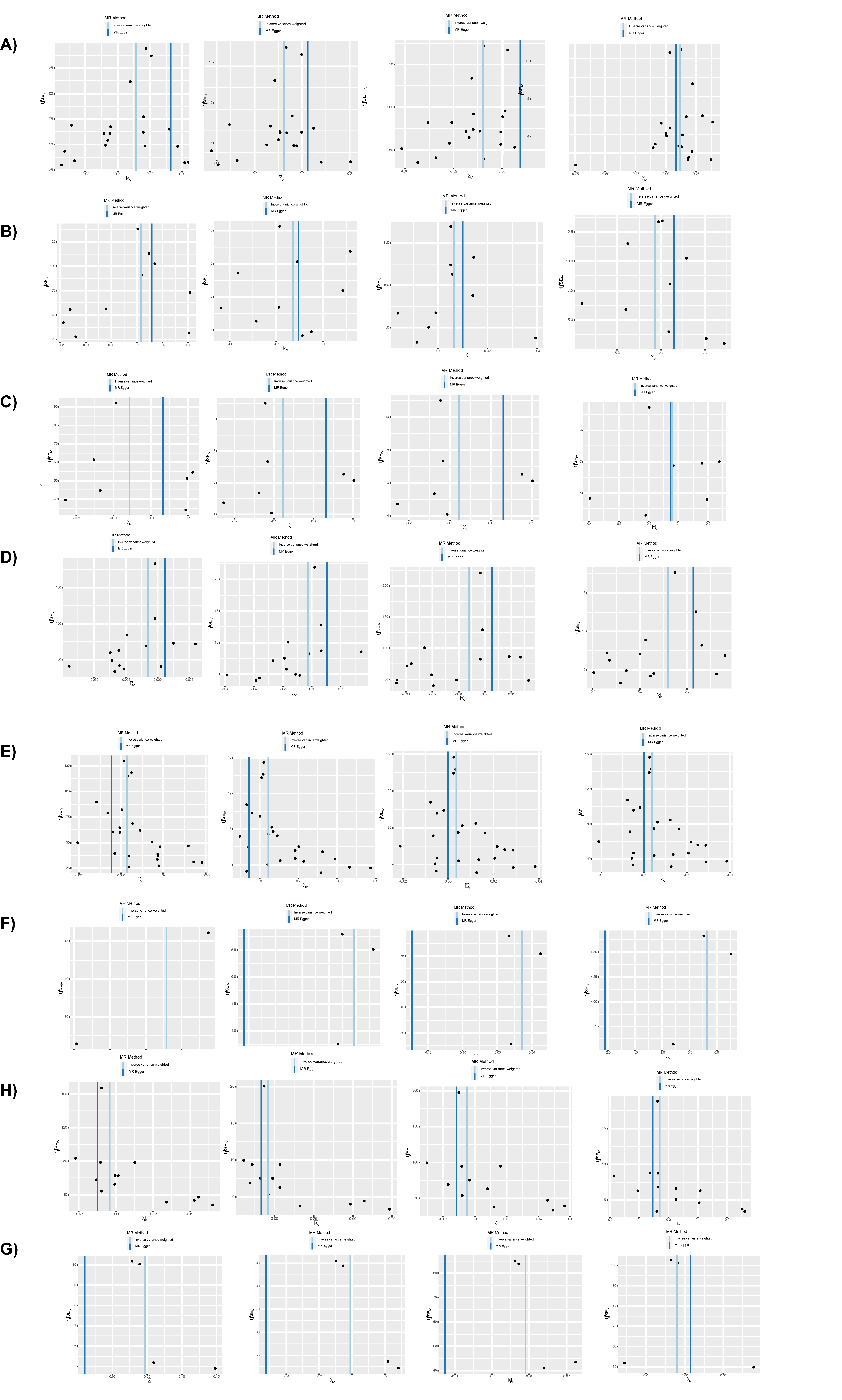

Supplement: Supplementary file 3 — Supplementary Material 3 [file 12967_2024_5359_MOESM3_ESM.png]

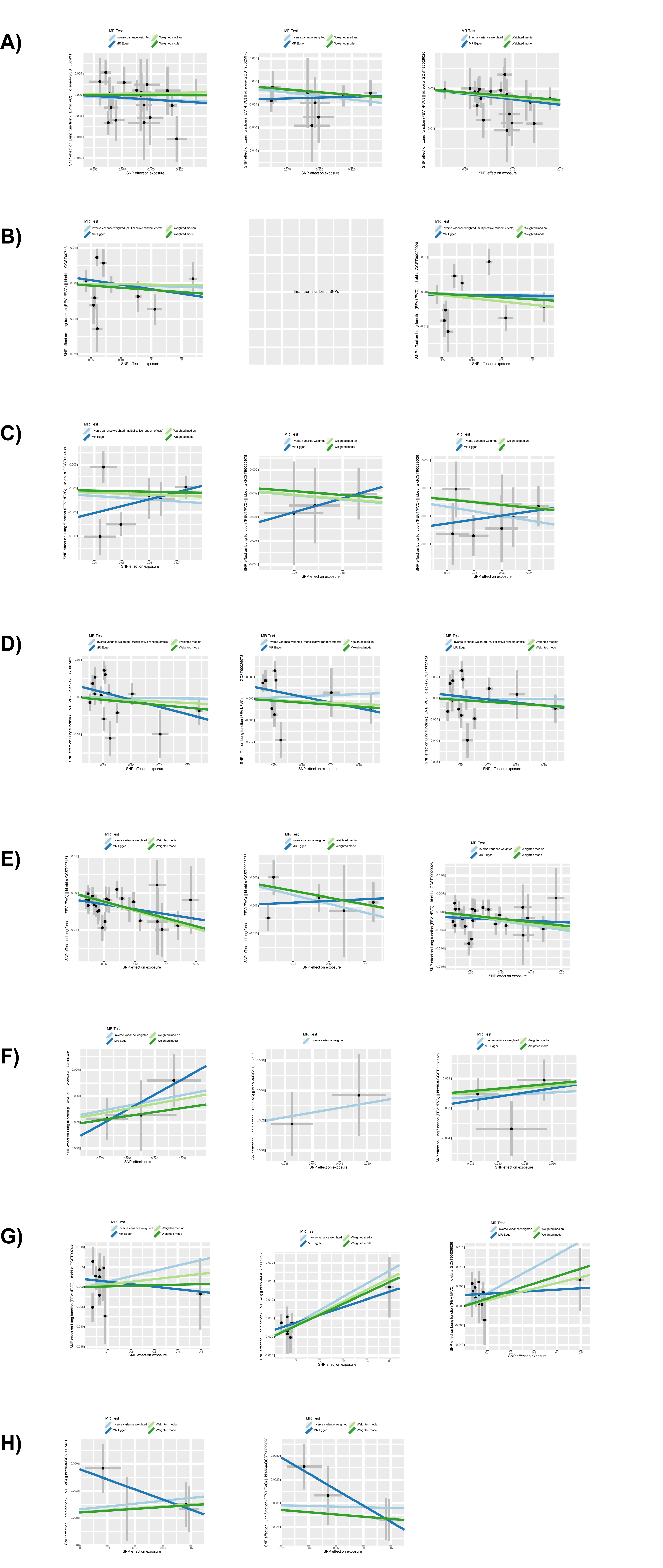

Supplement: Supplementary file 4 — Supplementary Material 4 [file 12967_2024_5359_MOESM4_ESM.png]

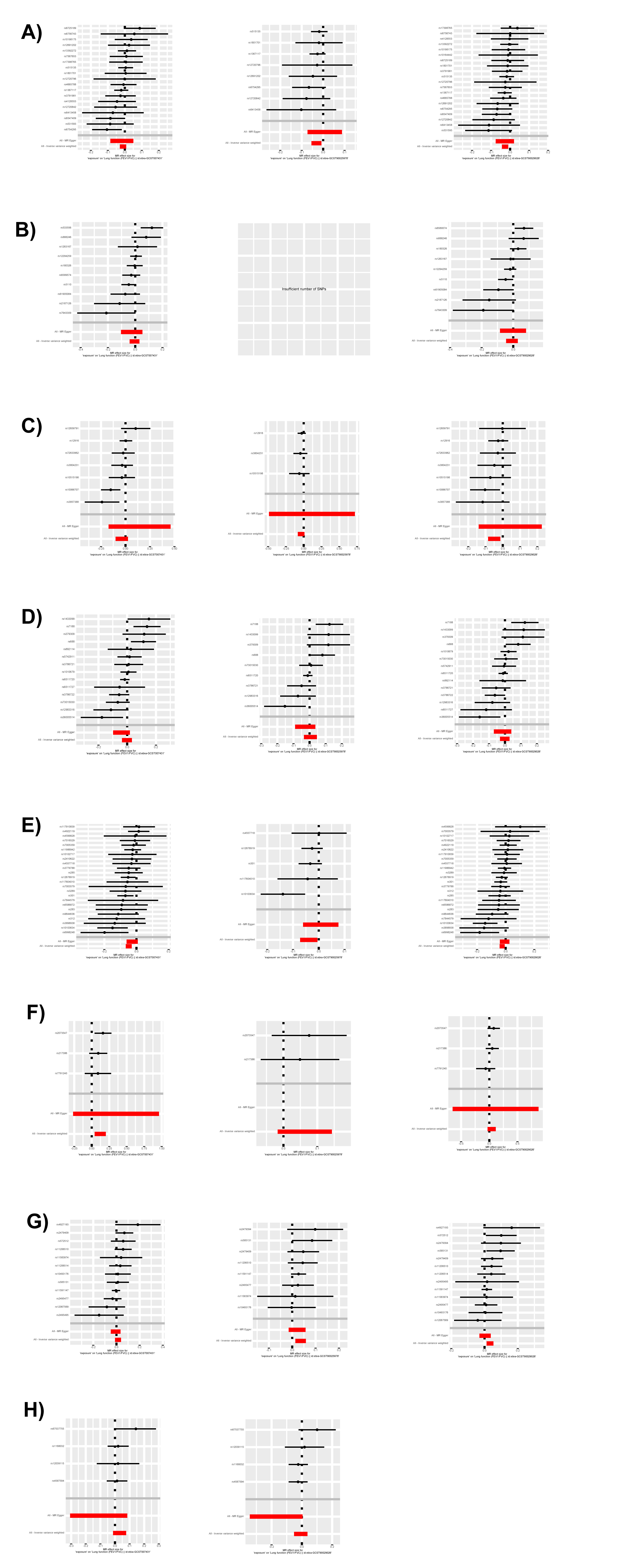

Supplement: Supplementary file 5 — Supplementary Material 5 [file 12967_2024_5359_MOESM5_ESM.png]

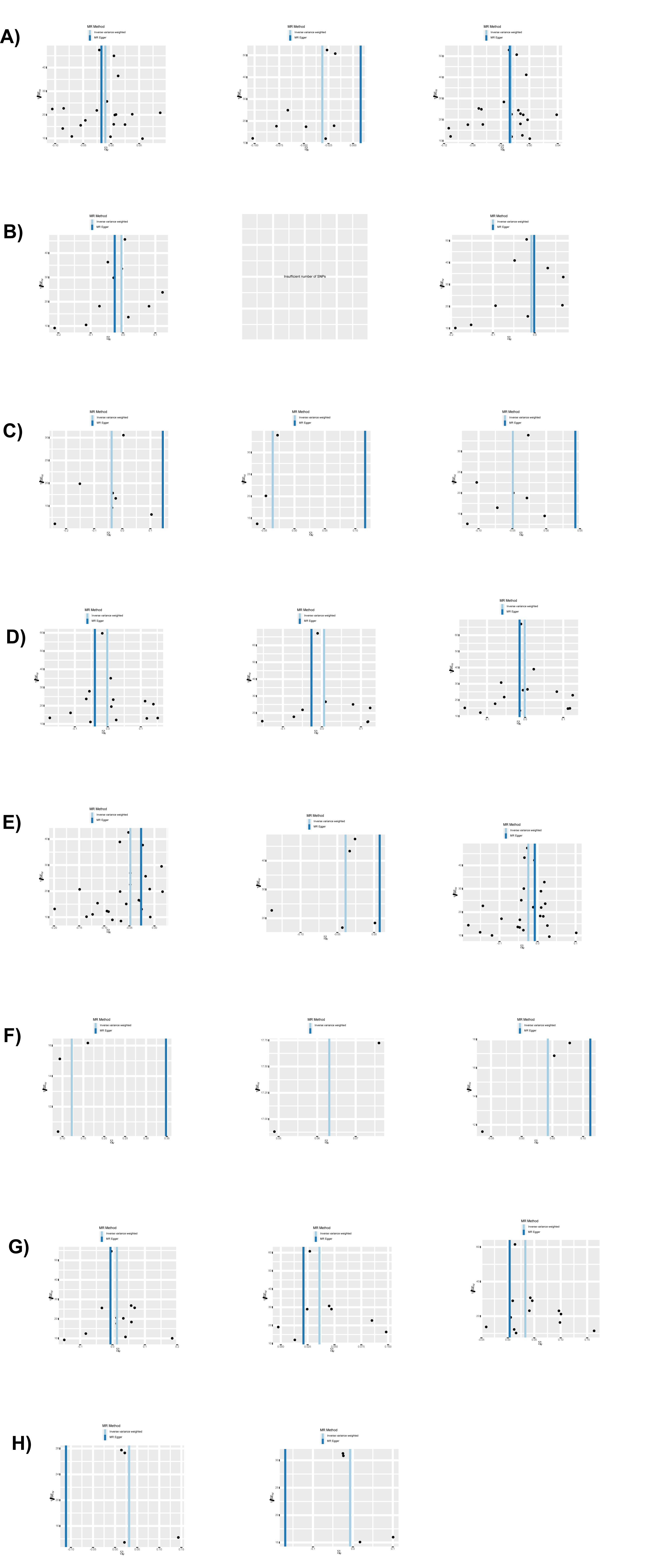

Supplement: Supplementary file 6 — Supplementary Material 6 [file 12967_2024_5359_MOESM6_ESM.png]

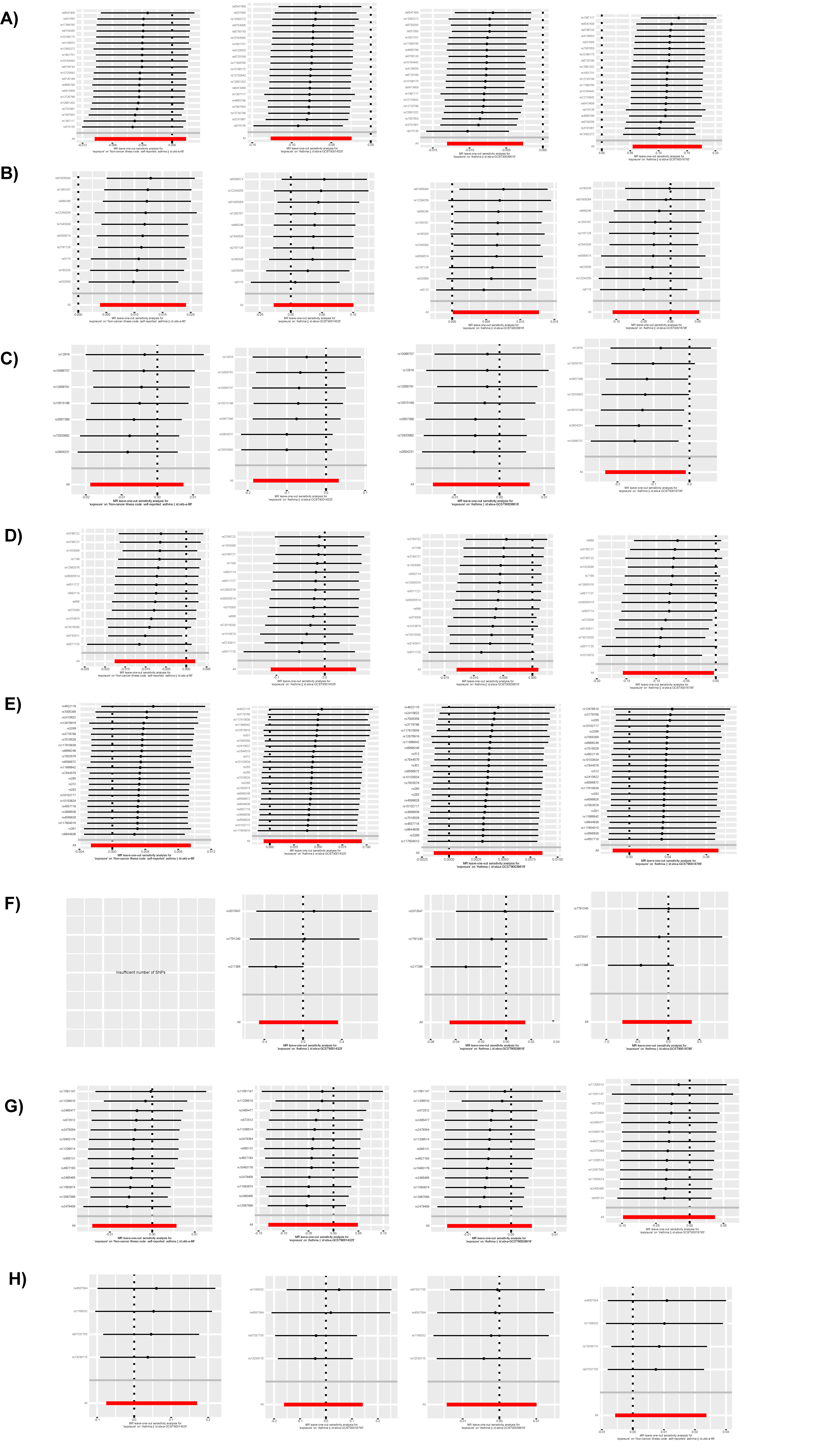

Supplement: Supplementary file 7 — Supplementary Material 7 [file 12967_2024_5359_MOESM7_ESM.png]

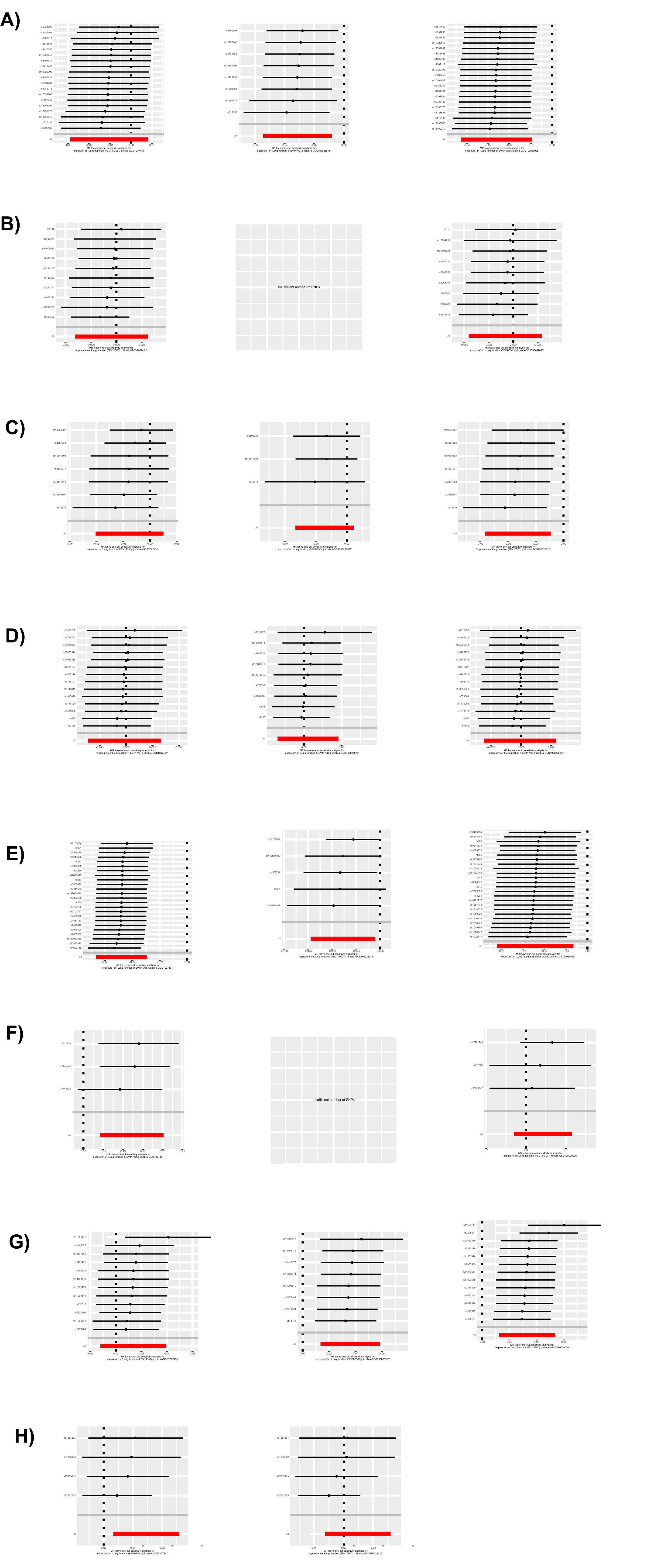

Supplement: Supplementary file 8 — Supplementary Material 8 [file 12967_2024_5359_MOESM8_ESM.png]
