# Supplementary material for: Repurposing lipid-lowering drugs on asthma and lung function: evidence from a genetic association analysis
Source: J Transl Med. 2024 Jul 3;22:615. doi: 10.1186/s12967-024-05359-5 (PMC11223406; doi:10.1186/s12967-024-05359-5)
Supplement: Supplementary file 9 — Supplementary Material 9 [file 12967_2024_5359_MOESM9_ESM.docx]

**Supplementary figure 1:** Scatter plot of the genetic association with lipid-lowering drugs against genetic association with asthma. (A: APOB, B: APOC3, C: HMGCR, D: LDLR, E: LPL, F: NPC1L1, G: PCSK9, H: ANGPTL3)

**Supplementary figure 2:** Forest plot of association of genetically lipid-lowering drugs targets with risk of asthma. (A: APOB, B: APOC3, C: HMGCR, D: LDLR, E: LPL, F: NPC1L1, G: PCSK9, H: ANGPTL3)

**Supplementary figure 3:** Plots of “leave-one-out” analyses for MR analyses of the causal effect of lipid-lowering drugs targets on asthma. (A: APOB, B: APOC3, C: HMGCR, D: LDLR, E: LPL, F: NPC1L1, G: PCSK9, H: ANGPTL3)

**Supplementary figure 4:** Funnel plot of the instrument strength against causal estimates of lipid-lowering drugs on asthma. (A: APOB, B: APOC3, C: HMGCR, D: LDLR, E: LPL, F: NPC1L1, G: PCSK9, H: ANGPTL3)

**Supplementary figure 5:** Scatter plot of the genetic association with lipid-lowering drugs against genetic association with lung function (FEV1/FVC). (A: APOB, B: APOC3, C: HMGCR, D: LDLR, E: LPL, F: NPC1L1, G: PCSK9, H: ANGPTL3)

**Supplementary figure 6:** Forest plot of association of genetically lipid-lowering drugs targets with risk of lung function (FEV1/FVC). (A: APOB, B: APOC3, C: HMGCR, D: LDLR, E: LPL, F: NPC1L1, G: PCSK9, H: ANGPTL3)

**Supplementary figure 7:** Plots of “leave-one-out” analyses for MR analyses of the causal effect of lipid-lowering drugs targets on lung function (FEV1/FVC). (A: APOB, B: APOC3, C: HMGCR, D: LDLR, E: LPL, F: NPC1L1, G: PCSK9, H: ANGPTL3)

**Supplementary figure 8:** Funnel plot of the instrument strength against causal estimates of lipid-lowering drugs on lung function (FEV1/FVC). (A: APOB, B: APOC3, C: HMGCR, D: LDLR, E: LPL, F: NPC1L1, G: PCSK9, H: ANGPTL3)
