# Supplementary material for: Repurposing lipid-lowering drugs on asthma and lung function: evidence from a genetic association analysis
Source: J Transl Med. 2024 Jul 3;22:615. doi: 10.1186/s12967-024-05359-5 (PMC11223406; doi:10.1186/s12967-024-05359-5)
Supplement: Supplementary file 10 — Supplementary Material 10 [file 12967_2024_5359_MOESM10_ESM.docx]

**Dear reviewers,**

**We appreciate your professional review, constructive comments, and valuable suggestions on our manuscript. We greatly appreciate the time and effort you have dedicated to reviewing our manuscript. Your feedback enhances the academic rigor of our article. We have made the necessary improvements to the manuscript as detailed below.**

**Reviewer #2:**

**2. "2. In Figure 4, what does 'lung function' refer to specifically?"**

**Minor: FEV1/FVC threshold for obstruction is 0.7 in COPD (not 0.8)**

**Response:** Thank you for bringing this to our attention. We apologize for the oversight in our previous response to the review comments, where "0.7" was incorrectly written as "0.8". We thank the reviewer for bringing this to our attention.

**3. Sensitivity analysis**

**Supplementary figures (esp. axis labels and legends) are very blurred in the downloaded word file.**

**Response:** Thanks for your suggestion. We are so sorry that the picture were be compressed in the “word document”. Hence, we will submit the original image file and the image legends with the new article modified version. We thank the reviewer for bringing this to our attention. Your valuable comments will make our articles more rigorous.

**7. Literature review expected for computational results of treating asthma with lipid-lowering drugs - seemed like there was no** **relevant literature from authors' response**

**Response:** We thank the reviewer for the opportunity to further clarify this important point. We apologize for not addressing this point in the revised manuscript. We conducted a literature search in “PubMed” and “Web of Science,” however, we were unable to find relevant literature. Until now, most researchers have studied the effects of single lipid-lowering drugs on asthma through retrospective clinical studies. For example, Park et al. found that statin use is associated with a lower risk of asthma exacerbation and better clinical outcomes in adult asthma patients [1]. Related research findings are detailed in “*Page 18, lines 319-342*.” However, it is not be reported to explore the relationship between asthma and lipid-lowering drugs using multi-omic-based and network-based approaches. Therefore, we intend to utilize multi-omic-based and network-based approaches in future studies to investigate the relationship between lipid-lowering drugs and asthma, with the aim of enhancing the reliability and robustness of our findings. We sincerely appreciate the valuable comments and guidance provided, which will inform our future research endeavors.

**Cite:** 1. Park C, Jang JH, Kim C, Lee Y, Lee E, Yang HM, Park RW, Park HS. Real-World Effectiveness of Statin Therapy in Adult Asthma. J Allergy Clin Immunol Pract. 2024 Feb;12(2):399-408.e6. doi: 10.1016/j.jaip.2023.10.029. Epub 2023 Oct 20. PMID: 37866433.
